# Genetic and Phylogenetic Characteristics of *Pasteurella multocida* Isolates From Different Host Species

**DOI:** 10.3389/fmicb.2018.01408

**Published:** 2018-06-26

**Authors:** Zhong Peng, Wan Liang, Fei Wang, Zhuofei Xu, Zhihao Xie, Zhenghan Lian, Lin Hua, Rui Zhou, Huanchun Chen, Bin Wu

**Affiliations:** ^1^Department of Preventive Veterinary Medicine, College of Veterinary Medicine, Huazhong Agricultural University, Wuhan, China; ^2^State Key Laboratory of Agricultural Microbiology, Huazhong Agricultural University, Wuhan, China; ^3^The Cooperative Innovation Center for Sustainable Pig Production, Huazhong Agricultural University, Wuhan, China; ^4^Hubei Key Laboratory of Animal Embryo and Molecular Breeding, Institute of Animal Husbandry and Veterinary Sciences, Hubei Academy of Agricultural Sciences, Wuhan, China; ^5^Ecological Research Institute, School of Life Science, South China Normal University, Guangzhou, China; ^6^Guangdong Magigene Biotechnology Co. Ltd., Guangzhou, China

**Keywords:** *Pasteurella multocida*, different host species, genetic characteristics, comparative genomics analysis, phylogeny

## Abstract

*Pasteurella multocida* is a leading cause of respiratory diseases in many host species. To understand the genetic characteristics of *P. multocida* strains isolated from different host species, we sequenced the genomic DNA of *P. multocida* isolated from pigs and analyzed the genetic characteristics of strains from avian species, bovine species, pigs, and rabbits using whole genome sequence (WGS) data. Our results found that a capsular: lipopolysaccharide (LPS): multilocus sequence typing (MLST) genotype A: L1: ST129 (43.75%) was predominant in avian *P. multocida*; while genotypes B: L2: ST122 (60.00%) and A: L3: ST79 (30.00%) were predominate in bovine *P. multocida*; genotype D: L6: ST50 (37.50%) in porcine *P. multocida*; and genotype A: L3: ST9 (76.47%) in rabbit *P. multocida*. Comparative genomic analysis of *P. multocida* from different host species found that there are no genes in the *P. multocida* genome that are specific to any type of host. Phylogenetic analysis using either whole-genome single nucleotide polymorphisms (SNPs) or the set of SNPs present in all single-copy core genes across genomes showed that *P. multocida* strains with the same LPS genotype and MLST genotype were clustered together, suggesting the combining both the LPS and MLST typing schemes better explained the topology seen in the *P. multocida* phylogeny.

## Introduction

*Pasteurella multocida* infects a wide spectrum of domestic and wild animals such as poultry and wild birds, pigs, cattle and buffaloes, rabbits, small ruminants, cats (including house cats and large wild cats), dogs and other mammals (Wilkie et al., 2012; Wilson and Ho, 2013). *P. multocida* isolates are associated with clinical manifestations ranging from asymptomatic or mild chronic upper respiratory inflammation to acute, pneumonic and/or disseminated disease (Wilson and Ho, 2013). While less common, *P. multocida* can also cause infections in humans through animal bites and/or scratches (Wilson and Ho, 2013).

There are many virulence factors contributing to the pathogenesis of *P. multocida*. Currently known virulence factors include genes involved in the formation of the capsule, lipopolysaccharide (LPS), fimbriae and adhesins, toxins, iron regulated and iron acquisition proteins, sialic acid metabolism, hyaluronidase, and outer membrane proteins (OMPs) (Harper et al., 2006). The two key surface components, capsule and LPS, form the main typing basis of *P. multocida*. Serologically, the bacterium is classified into five capsular serogroups (A, B, D, E, F) and/or 16 somatic serotypes according to its capsule and/or LPS antigens, respectively (Carter, 1955; Heddleston et al., 1972). These traditional serological typing methods are too complicated to be used, because the preparation of the high-sensitive anti-serum required for these methods is very difficult (Peng et al., 2016). Therefore, molecular typing methods have been developed to help assign *P. multocida* into five capsular genotypes (A, B, D, E, F) (Townsend et al., 2001) and eight LPS genotypes (L1-L8) (Harper et al., 2015). In addition, the multilocus sequence typing method has been also designed for *P. multocida* (Subaaharan et al., 2010), and it has been widely used in epidemiology and surveillance (Hotchkiss et al., 2011; Moustafa et al., 2013; Fernández-Rojas et al., 2014; Peng et al., 2018).

It is documented that the infection of *P. multocida* isolates displays host predilection (Wilkie et al., 2012; Wilson and Ho, 2013). For example, a *P. multocida* B: 2 strain can kill cattle and buffaloes at a low dose, but it has no effect on chickens, even at very high doses (Aktories et al., 2012). Isolates from non-avian hosts generally do not cause symptoms of fowl cholera in birds (Ahir et al., 2011; Peng et al., 2017). However, little is known about the genetic characteristics of *P. multocida* strains circulating in different hosts. In this study, the genomic DNA of *P. multocida* strains isolated from pigs was sequenced, and the genetic characteristics of *P. multocida* isolates from avian hosts, bovine hosts, pigs and rabbits were determined using the previously published whole genome sequences (WGSs) data. The aim of this study is to understand the genetic characteristics of *P. multocida* strains circulating in different host species.

## Materials and methods

### *P. multocida* strains and whole genome sequencing

A total of 47 *P. multocida* strains were selected for whole genome sequencing in this study (Table 1). Most of these strains were isolated from pigs with respiratory disorders in China (Peng et al., 2018), with the exception of strain HN04, which is a capsular type B isolate from a swine haemorrhagic septicaemia case. The capsular types and LPS genotypes of these 47 *P. multocida* isolates were determined through PCR assays, as described previously (Townsend et al., 2001; Harper et al., 2015).

**Table 1:** *Pasteurella multocida* strains discussed in this study.

**Strain**	**Host**	**Size (Mb)**	**GC%**	**Contigs**	**CDS**	**tRNA**	**rRNA**	**Capsular genotypes**	**LPS genotypes**	**MLST genotypes**	**Clonal complex**	**GenBank accession**
ATCC 11039	Avian	2.27	40.3	19	2040	50	9	A	L1	ST60	ST158	LUDA00000000
ATCC 15742	Avian	2.31	40.3	17	2084	50	9	A	L3	ST8	ST8	LUDB00000000
ATCC 1662	Avian	2.27	40.3	16	2028	50	9	A	L3	ST53	Not defined	LUDC00000000
ATCC 1702	Avian	2.32	40.3	25	2065	50	10	A	L2	ST156	ST156	LUDD00000000
ATCC 2095	Avian	2.32	40.2	16	2092	54	8	A	L5	ST159	Not defined	LUDE00000000
ATCC 2100	Avian	2.39	40.1	41	2180	49	8	A	L6	ST27	ST74	LUDF00000000
C48-1	Avian	2.26	40.2	17	2098	51	9	A	L1	ST129	ST129	LODS00000000
DY120818	Avian	2.28	40.3	24	2053	52	9	A	L1	ST129	ST129	LUCZ00000000
GX-Pm	Avian	2.29	40.3	8	1294	52	12	A	L1	ST129	ST129	JZXO00000000
HB02	Avian	2.21	40.3	46	1986	45	6	A	L1	ST129	ST129	LYOX00000000
HN141014	Avian	2.28	40.3	25	2055	52	10	A	L1	ST129	ST129	LUCX00000000
P1059	Avian	2.31	40.3	39	2101	50	4	A	L3	ST8	ST8	CM001581
Pm70	Avian	2.26	40.4	1	2021	56	19	F	L3	ST9	ST9	AE004439
Razi 0002	Avian	2.29	40.3	1	1903	56	19	A	L1	ST129	ST129	CP019081
RCAD0276	Avian	2.22	40.3	16	1983	50	9	A	L1	ST129	ST129	LUCY00000000
X73	Avian	2.27	40.3	24	2058	50	4	A	L1	ST60	ST158	CM001580
Avian *P. multocida* average		2.28	40.3	21	2003	51	10	–	–	–	–	–
36950	Bovine	2.35	40.4	1	2064	53	19	A	L3	ST79	ST13	CP003022
2125PM	Bovine	2.30	40.3	29	2061	45	4	A	L3	ST79	ST13	LQCZ00000000
2612PM	Bovine	2.30	40.3	27	2063	44	5	A	L3	ST79	ST13	LQEQ00000000
2887PM	Bovine	2.42	40.2	40	2219	47	6	A	L3	ST79	ST13	LQFA00000000
BUKK	Bovine	2.36	40.4	37	2153	50	4	B	L2	ST122	ST122	JQAO00000000
HB01	Bovine	2.42	40.3	1	2196	55	19	A	L3	ST79	ST13	CP006976
HS SKN01	Bovine	2.45	40.7	48	2149	43	10	B	L2	ST122	ST122	MDXJ00000000
Islm	Bovine	2.35	40.3	32	2142	51	4	B	L2	ST122	ST122	JQAB00000000
P1062	Bovine	2.70	40.7	5	2296	53	19	A	L3	ST80	ST13	CM002276
Pesh	Bovine	2.35	40.3	32	2144	51	4	B	L2	ST122	ST122	JQAC00000000
Pm-3	Bovine	2.42	40.3	1	2202	55	19	A	L3	ST79	ST13	CP014618
PMTB	Bovine	2.20	40.4	8	2021	51	10	B	L2	ST122	ST122	AWTD00000000
PVAcc	Bovine	2.35	40.3	38	2141	50	4	B	L2	ST122	ST122	JQAD00000000
Razi_Pm0001	Bovine	2.36	40.4	1	2053	59	19	B	L2	ST122	ST122	CP017961
THA	Bovine	2.30	40.4	29	2082	50	4	B	L2	ST122	ST122	JQAE00000000
THD	Bovine	2.30	40.4	28	2082	50	4	B	L2	ST122	ST122	JQAF00000000
THF	Bovine	2.30	40.4	28	2082	51	4	B	L2	ST122	ST122	JQAG00000000
TX1	Bovine	2.41	40.3	35	2179	50	4	B	L2	ST122	ST122	JQAH00000000
V1	Bovine	2.35	40.3	27	2137	50	4	B	L2	ST122	ST122	JQAI00000000
ATCC 51689	Bovine	2.27	40.3	16	2098	46	14	A	L1	ST65	Not defined	FOWO00000000
Bovine *P. multocida* average		2.36	40.4	23	2128	50	9	–	–	–	–	–
3480	Porcine	2.38	40.3	1	2127	54	19	A	L6	ST74	ST74	CP001409
ATCC 43137	Porcine	2.27	40.4	1	2021	59	17	A	L3	ST13	ST13	CP008918
HB03	Porcine	2.31	40.4	1	2050	53	19	A	L3	ST13	ST13	CP003328
HN06	Porcine	2.41	40.2	1	2202	54	19	D	L6	ST50	ST50	CP003313
HN07	Porcine	2.33	40.3	1	2098	53	19	F	L3	ST9	ST9	CP007040
NCTC10322	Porcine	2.27	40.4	1	2025	56	19	A	L3	ST13	ST13	LT906458
SH02	Porcine	2.36	40.4	29	2138	48	1	D	L6	ST50	ST50	NWTL00000000
SH03	Porcine	2.46	40.1	28	2244	45	4	A	L6	ST74	ST74	NWTS00000000
SH05	Porcine	2.44	39.7	36	2245	56	1	F	L3	ST9	ST9	NWTT00000000
**HN04**	**Porcine**	**2.22**	**40.3**	**103**	**2045**	**43**	**3**	**B**	**L2**	**ST122**	**ST122**	**PPVE00000000**
**HN05**	**Porcine**	**2.32**	**40.2**	**51**	**2150**	**54**	**3**	**D**	**L6**	**ST50**	**ST50**	**PPVF00000000**
**HNA01**	**Porcine**	**2.28**	**40.5**	**29**	**2066**	**51**	**9**	**A**	**L3**	**ST7**	**Not defined**	**PPVG00000000**
**HNA02**	**Porcine**	**2.34**	**40.2**	**18**	**2168**	**49**	**9**	**A**	**L6**	**ST74**	**ST74**	**PPVH00000000**
**HNA03**	**Porcine**	**2.31**	**40.4**	**66**	**2122**	**50**	**7**	**A**	**L3**	**ST13**	**ST13**	**PPVI00000000**
**HNA04**	**Porcine**	**2.37**	**40.2**	**32**	**2214**	**48**	**10**	**A**	**L6**	**ST74**	**ST74**	**PPVJ00000000**
**HNA05**	**Porcine**	**2.38**	**40.2**	**19**	**2223**	**48**	**10**	**A**	**L6**	**ST74**	**ST74**	**PPVK00000000**
**HNA06**	**Porcine**	**2.43**	**40.2**	**36**	**2275**	**50**	**9**	**A**	**L6**	**ST27**	**ST74**	**PPVL00000000**
**HNA07**	**Porcine**	**2.35**	**40.2**	**19**	**2178**	**48**	**10**	**A**	**L6**	**ST74**	**ST74**	**PPVM00000000**
**HNA08**	**Porcine**	**2.36**	**40.2**	**29**	**2199**	**50**	**7**	**A**	**L3**	**ST13**	**ST13**	**PPVN00000000**
**HNA09**	**Porcine**	**2.33**	**40.3**	**26**	**2173**	**50**	**8**	**A**	**L3**	**ST13**	**ST13**	**PPVO00000000**
**HNA10**	**Porcine**	**2.42**	**40.1**	**23**	**2266**	**51**	**10**	**A**	**L6**	**ST74**	**ST74**	**PPVP00000000**
**HNA11**	**Porcine**	**2.40**	**40.2**	**30**	**2238**	**48**	**10**	**A**	**L6**	**ST74**	**ST74**	**PPVQ00000000**
**HNA12**	**Porcine**	**2.35**	**40.2**	**20**	**2179**	**49**	**10**	**A**	**L6**	**ST74**	**ST74**	**PPVR00000000**
**HNA13**	**Porcine**	**2.32**	**40.3**	**31**	**2156**	**49**	**7**	**A**	**L3**	**ST13**	**ST13**	**PPVS00000000**
**HNA14**	**Porcine**	**2.35**	**40.3**	**30**	**2210**	**50**	**7**	**A**	**L3**	**ST13**	**ST13**	**PPVT00000000**
**HNA15**	**Porcine**	**2.35**	**40.3**	**29**	**2210**	**50**	**7**	**A**	**L3**	**ST13**	**ST13**	**PPVU00000000**
**HNA16**	**Porcine**	**2.35**	**40.2**	**18**	**2175**	**49**	**9**	**A**	**L6**	**ST74**	**ST74**	**PPVV00000000**
**HNA17**	**Porcine**	**2.37**	**40.2**	**30**	**2214**	**50**	**7**	**A**	**L3**	**ST13**	**ST13**	**PPVW00000000**
**HNA18**	**Porcine**	**2.37**	**40.2**	**29**	**2205**	**48**	**8**	**A**	**L3**	**ST13**	**ST13**	**PPVX00000000**
**HNA19**	**Porcine**	**2.37**	**40.2**	**32**	**2206**	**48**	**9**	**A**	**L3**	**ST13**	**ST13**	**PPVY00000000**
**HNA20**	**Porcine**	**2.37**	**40.2**	**29**	**2205**	**48**	**9**	**A**	**L3**	**ST13**	**ST13**	**PPVZ00000000**
**HNA21**	**Porcine**	**2.42**	**40.1**	**44**	**2273**	**51**	**10**	**A**	**L6**	**ST74**	**ST74**	**PPWA00000000**
**HNA22**	**Porcine**	**2.42**	**40.1**	**43**	**2272**	**51**	**10**	**A**	**L6**	**ST74**	**ST74**	**PPWB00000000**
**HND01**	**Porcine**	**2.40**	**40.1**	**51**	**2249**	**53**	**8**	**D**	**L6**	**ST50**	**ST50**	**PPWC00000000**
**HND02**	**Porcine**	**2.34**	**40.3**	**41**	**2191**	**51**	**7**	**D**	**L6**	**ST287**	**Not defined**	**PPWD00000000**
**HND03**	**Porcine**	**2.38**	**40.0**	**35**	**2230**	**53**	**7**	**D**	**L6**	**ST50**	**ST50**	**PPWE00000000**
**HND04**	**Porcine**	**2.37**	**40.2**	**31**	**2213**	**49**	**8**	**D**	**L6**	**ST50**	**ST50**	**PPWF00000000**
**HND05**	**Porcine**	**2.29**	**40.3**	**23**	**2141**	**50**	**8**	**D**	**L6**	**ST50**	**ST50**	**PPWG00000000**
**HND06**	**Porcine**	**2.32**	**40.2**	**48**	**2166**	**53**	**8**	**D**	**L6**	**ST50**	**ST50**	**PPWH00000000**
**HND07**	**Porcine**	**2.35**	**40.2**	**34**	**2186**	**52**	**8**	**D**	**L6**	**ST50**	**ST50**	**PPWI00000000**
**HND08**	**Porcine**	**2.30**	**40.2**	**35**	**2133**	**50**	**8**	**D**	**L6**	**ST50**	**ST50**	**PPWJ00000000**
**HND09**	**Porcine**	**2.39**	**40.7**	**28**	**2190**	**51**	**8**	**D**	**L6**	**ST50**	**ST50**	**PPWK00000000**
**HND10**	**Porcine**	**2.39**	**40.2**	**48**	**2236**	**53**	**8**	**D**	**L6**	**ST50**	**ST50**	**PPWL00000000**
**HND11**	**Porcine**	**2.40**	**40.6**	**24**	**2198**	**51**	**9**	**D**	**L6**	**ST50**	**ST50**	**PPWN00000000**
**HND12**	**Porcine**	**2.22**	**40.3**	**20**	**2029**	**50**	**9**	**D**	**L6**	**ST287**	**Not defined**	**PPWM00000000**
**HND13**	**Porcine**	**2.22**	**40.3**	**20**	**2027**	**50**	**9**	**D**	**L6**	**ST287**	**Not defined**	**PPWO00000000**
**HND14**	**Porcine**	**2.40**	**40.7**	**31**	**2201**	**50**	**8**	**D**	**L6**	**ST50**	**ST50**	**PPWP00000000**
**HND15**	**Porcine**	**2.31**	**40.3**	**32**	**2147**	**49**	**8**	**D**	**L6**	**ST50**	**ST50**	**PPWQ00000000**
**HND16**	**Porcine**	**2.35**	**40.3**	**39**	**2214**	**51**	**9**	**D**	**L6**	**ST50**	**ST50**	**PPWR00000000**
**HND17**	**Porcine**	**2.30**	**40.3**	**36**	**2124**	**52**	**8**	**D**	**L6**	**ST50**	**ST50**	**PPWS00000000**
**HND18**	**Porcine**	**2.47**	**40.7**	**33**	**2282**	**51**	**8**	**D**	**L6**	**ST50**	**ST50**	**PPWT00000000**
**HND19**	**Porcine**	**2.27**	**40.2**	**22**	**2101**	**50**	**8**	**D**	**L6**	**ST50**	**ST50**	**PPWU00000000**
**HND20**	**Porcine**	**2.50**	**40.6**	**41**	**2330**	**51**	**9**	**D**	**L6**	**ST50**	**ST50**	**PPWV00000000**
**HND21**	**Porcine**	**2.37**	**40.2**	**34**	**2213**	**46**	**9**	**D**	**L6**	**ST50**	**ST50**	**PPWW00000000**
**HNF01**	**Porcine**	**2.28**	**40.3**	**20**	**2093**	**50**	**10**	**F**	**L3**	**ST9**	**ST9**	**PPWX00000000**
**HNF02**	**Porcine**	**2.27**	**40.3**	**19**	**2094**	**50**	**10**	**F**	**L3**	**ST9**	**ST9**	**PPWY00000000**
Porcine *P. multocida* average		2.35	40.3	28	2177	50	9	–	–	–	–	–
CIRMBP-0747	Rabbit	2.27	40.2	16	2078	46	4	A	L3	ST9	ST9	MTIE00000000
CIRMBP-0749	Rabbit	2.34	40.3	15	2160	45	4	A	L3	ST204	ST9	MTEA00000000
CIRMBP-0758	Rabbit	2.41	40.2	30	2237	45	–	A	L6	ST298	Not defined	MTEB00000000
CIRMBP-0760	Rabbit	2.40	40.2	31	2220	45	9	A	L6	ST298	Not defined	MTEC00000000
CIRMBP-0782	Rabbit	2.27	40.2	12	2075	47	3	A	L3	ST9	ST9	MTED00000000
CIRMBP-0783	Rabbit	2.33	40.3	16	2155	47	–	A	L3	ST204	ST9	MTEF00000000
CIRMBP-0812	Rabbit	2.34	40.3	16	2158	47	4	A	L3	ST204	ST9	MTDZ00000000
CIRMBP-0817	Rabbit	2.43	40.2	38	2271	50	–	A	L6	ST74	ST74	MTEE00000000
CIRMBP-0827	Rabbit	2.37	40.2	36	2205	49	4	A	L3	ST204	ST9	MTIG00000000
CIRMBP-0835	Rabbit	2.27	40.2	21	2080	49	4	A	L3	ST9	ST9	MTIF00000000
CIRMBP-0872	Rabbit	2.21	40.3	15	1998	47	5	A	L3	ST9	ST9	MTIH00000000
CIRMBP-0873	Rabbit	2.48	40.4	1	2152	57	19	A	L3	ST9	ST9	CP020347
CIRMBP-0877	Rabbit	2.35	40.2	29	2171	49	2	D	L6	ST50	ST50	MTII00000000
CIRMBP-0884	Rabbit	2.46	40.5	1	2210	54	19	A	L3	ST9	ST9	CP020345
CIRMBP-0906	Rabbit	2.27	40.2	15	2080	47	–	A	L3	ST9	ST9	MTIJ00000000
CIRMBP-0922	Rabbit	2.28	40.2	14	2083	48	6	A	L3	ST9	ST9	MTIK00000000
CIRMBP-0927	Rabbit	2.28	40.2	20	2089	49	–	A	L3	ST9	ST9	MTIL00000000
Rabbit *P. multocida* average		2.34	40.3	19	2142	48	7	–	–	–	–	–

*Strains marked in blue are the 47 porcine P. multocida strains sequenced in this study, and strains marked in black are retrieved from NCBI Genome database (https://www.ncbi.nlm.nih.gov/genome/genomes/912)*.

The genomic DNA (gDNA) of the 47 *P. multocida* strains were isolated using a QIAGEN Blood and Cell Culture DNA Midi Kit (Lot NO. 157037192, QIAGEN, Germany) with QIAGEN Genomic-tip 100/G (Lot NO. 157024372, QIAGEN, Germany). DNA quantity and quality were evaluated by electrophoresis on a 1% agarose gel and evaluation using a NanoDrop2000 (Thermo Scientific, Waltham, USA), respectively.

DNA libraries were constructed using NEBNext®Ultra™ II DNA Library Prep Kit. Sequencing was carried out on an Illumina Hiseq Xten platform (Illumina Inc., San Diego, USA) at Guangdong Magigene Biotechnology Co. LTD (Guangzhou, China), using the pair-end 150-bp sequencing protocol. The strategy yielded 5,308,336~12,159,104 raw reads. The raw reads were then filtered to eliminate reads with low quality according to the following criteria: low quality base pairs at each terminal of the reads (Quality-Value < 20) were removed; reads with over short length (parameter setting at 50 bp), > 10% Ns (parameter setting at 10 bp) or > 15 bp overlap overlap with Illumina TruSeq adapter sequences (parameter setting at 15 bp) were removed. After that, 4,732,078~10,715,272 clean reads (Q20% = 100, Q30% ≥ 95.64) were produced. High quality reads were *de novo* assembled via SPAdes v3.9.0 (Bankevich et al., 2012) to generate contigs.

In addition to the 47 porcine *P. multocida* sequenced herein, we also retrieved genome sequences of *P. multocida* from different hosts from the National Center for Biotechnology Information (NCBI) genome database (https://www.ncbi.nlm.nih.gov/genome/genomes/912). By the time of this study design (Assessed at December 31, 2017), there are 132 *P. multocida* genome sequences publicly available. To ensure the reliability and quality of our analysis, only those high-quality genomes assemblies with contigs numbers ≤ 50 plus coverage ≥50 × were selected. While not meeting the criterion of selection, the two avian *P. multocida* isolates P1059 (GenBank accession number: CM001581) and X73 (GenBank accession number: CM001580) were still included, because their phenotypical and genetic characteristics have been published (Johnson et al., 2013). The strategy resulted in 62 *P. multocida* genome sequences, which include 16 avian, 20 bovine, 9 porcine, and 17 rabbit isolates (Table 1). A total of 109 *P. multocida* genome sequences were finally used in this study, including 47 sequenced herein plus 62 retrieved from NCBI database (Table 1).

Draft genome sequences of the 47 porcine *P. multocida* were annotated using prokka v1.12 (Seemann, 2014) following the NCBI Prokaryotic Genome Annotation Pipeline (Angiuoli et al., 2008). The 47 annotated genomes were submitted to GenBank accession numbers are displayed in Table 1, which are highlighted in blue text.

### Genotyping

The capsular genotypes and LPS genotypes of the 47 *P. multocida* strains sequenced were determined using PCR methods (Townsend et al., 2001; Harper et al., 2015) and are documented on our previous publication (Peng et al., 2018). Capsular genotypes and LPS genotypes of the 62 *P. multocida* genomes from NCBI genome database were determined by blasting the oligonucleotide sequences designed for capsular typing (Townsend et al., 2001) and LPS genotyping (Harper et al., 2015) against their WGSs, respectively. MLST genotypes of the 109 *P. multocida* strains were assigned by performing blast of their WGSs against the *P. multocida* MLST Databases (https://pubmlst.org/pmultocida). Currently, there are two separate *P. multocida* MLST schemes on the database, i.e., Multi-host MLST and RIRDC MLST. As for some bovine isolates (BUKK, Islm, Pesh, PVAcc, THA, THD, THF, TX1, V1) included in this study, an existing study has reported the use of RIRDC MLST in determining their MLST genotypes (Moustafa et al., 2013), we use the same MLST scheme to determine the *P. multocida* MLST genotypes in this study.

### Phylogenetic trees

The phylogenetic relationship between *P. multocida* strains from different host species was predicted by analyzing the whole-genome single nucleotide polymorphisms (WG-SNPs) as well as the set of SNPs present in all single-copy core genes across genomes (CG-SNPs). The WG-SNPs were identified by comparing each of the WGSs against the reference *P. multocida* ATCC 43137 genome sequence (GenBank accession NO. CP008918) using MAFFT (version 7.222) software (Katoh and Standley, 2013). A phylogenetic tree based on these WG-SNPs was exported using Parsnp (version 1.2) software (Treangen et al., 2014). Another phylogenetic tree based on CG-SNPs was also constructed. In this strategy, gene contents of each of the 109 *P. multocida* genomes were clustered using Roary v3.11.0 software (Page et al., 2015) with a criterion of 90% DNA identity plus 90% gene coverage as the minimum criteria for a match to define core genes at first, then SNPs within all the single-copy core genes were compared to generate a neighbor-joining tree using MEGA6 with Kimura 2-parameter model and 1000 bootstrap (Tamura et al., 2013).

### Core and pan-genome analysis

Gene contents of each of the 109 *P. multocida* genomes were clustered using Roary v3.11.0 (Page et al., 2015). Homologous genes were identified by using 90% DNA identity and 90% gene coverage as the minimum criteria for a match. Because potential genes might be disrupted by the gaps between the contigs, we defined those genes present in 99~100% of the strains as core genes. Dispensable genes were defined as those genes shared by at least two strains but shared by <99% strains, while unique genes were defined those present only in a single strain. Host-specific genes were defined as those genes shared by all strains isolated from the same host species but absent in the strains from the other host species. Core genes, dispensable genes, and strain-specific were annotated using the Clusters of Orthologous Groups of proteins (COGs) database (http://www.ncbi.nlm.nih.gov/COG/) for functional analysis (Galperin et al., 2015).

### Distribution of main virulence factors associated genes

Genes identified as virulence factors (VFGs) were predicted by performing BLAST analysis of the genome sequence against the bacterial Virulence Factor Database (VFDB) (Chen et al., 2005, 2012). After that, a BLAST score ratio (BSR) analysis was performed on these predicted VFGs, and the presence of a VFG in a genome was determined based upon the BSR analysis with a normalized ratio ≥0.8, as described previously (Rasko et al., 2008). A heat-map showing the BSR ratio values of each gene across the genomes of all bacterial strains was generated.

## Results

### General features of the 47 porcine *P. multocida* genomes

Whole genome sequencing yielded approximately 796.25~1823.87 Mbp raw data for the 47 porcine *P. multocida* isolates. The data filter strategy produced approximately 701.86~1589.23 Mb clean data for assembly. Sequences assembled using SPAdes v3.9.0 (Bankevich et al., 2012) generated approximately 18~66 contigs for the 47 porcine *P. multocida* isolates, with an average of 25 contigs for each strain. These contigs represented a 161~631-kb N50 fragment with an average contig size of approximately 35~130 kb. The predicted genome sizes ranged from 2.22 to 2.49 Mbp, with average G+C content ranging from 40.04 to 40.67% for the 47 *P. multocida* isolates. The average genome size and G+C content for each of the *P. multocida* sequenced were 2.35 Mbp and 40.3%, respectively. The number of coding DNA sequences among the genome sequences ranged from 2,027 to 2,330. The general features of the 47 sequenced *P. multocida* isolates from pigs and their accession numbers as well as those public available genomes of *P. multocida* from different hosts are shown in Table 1.

### Genotypical characteristics of the *P. multocida* isolates from different hosts

#### Capsular genotypes

Capsular typing strategy identified two categories of capsular genotypes (A and F) for the 16 avian *P. multocida* isolates, two categories of capsular genotypes (A and B) for the 20 bovine *P. multocida* isolates, four categories of capsular genotypes (A, B, D, and F) for the 56 porcine *P. multocida* isolates, and two categories of capsular genotypes (A and D) for the 17 rabbit *P. multocida* isolates. Type A was the most common capsular genotype for the avian *P. multocida* isolates (93.75% of the isolates) and rabbit *P. multocida* isolates (94.12% of the isolates); types A and B were the common capsular genotypes for the bovine *P. multocida* isolates (40.00% of the isolates and 60.00% of the isolates, respectively); types A and D were the common capsular genotypes for the porcine *P. multocida* isolates (48.21% of the isolates and 42.86% of the isolates, respectively; see Table 1 and Image 1 in Supplementary Materials).

#### LPS genotypes

LPS typing strategy identified five categories of LPS genotypes (L1, L2, L3, L5, and L6) for the 16 avian *P. multocida* isolates. L1 (56.25%) was the dominant genotype with the highest percentage, followed by L3 (25.00%). For the 20 bovine *P. multocida* isolates, three categories of LPS genotypes (L1, L2, L3) were identified, and L2 (60.00%) was the mostly common LPS genotypes, followed by L3 (35.00%). There were also three categories of LPS genotypes (L2, L3, L6) defined for the 56 porcine *P. multocida* isolates. Among them, L6 (66.07%) ranked the highest position, followed by L3 (32.14%). For the 17 rabbit *P. multocida* isolates, two categories of LPS genotypes (L3 and L6) were identified, and L3 (76.47%) was the most cited (see Table 1 and Image 2 in Supplementary Materials).

**Figure 1:**
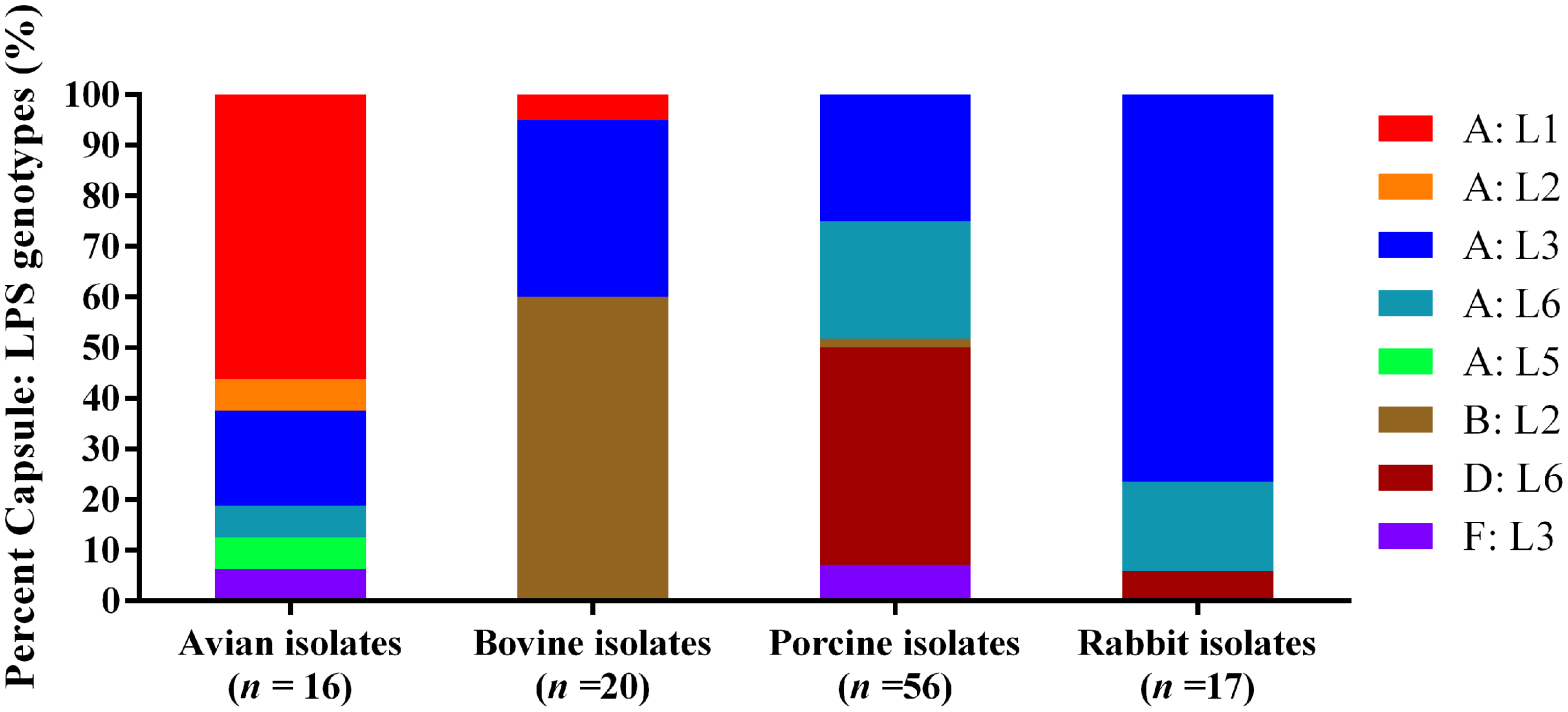
Distribution of different capsule: LPS genotypes in *P. multocida* isolated from different hosts.

When combining capsular types with LPS genotypes, the mostly commonly identified capsule: LPS genotype for the 16 avian isolates was A: L1 (56.25%), followed by A: L3 (18.75%). For the 20 bovine isolates, type B: L2 (60.00%) was the most common, followed by A: L3 (35.00%). Types D: L6 (42.86%), A: L3 (25.00%), and A: L6 (23.21%) were the dominant capsule: LPS genotypes for the 56 porcine isolates, and A: L3 (76.47%) was the most dominant capsule: LPS genotypes for the 17 rabbit isolates (Figure 1).

#### MLST genotypes

RIRDC MLST assigned eight categories of MLST genotypes (ST8, ST9, ST27, ST53, ST60, ST129, ST156, ST159) for the 16 avian *P. multocida*, and ST129 (43.75%) was the predominate MLST genotype. The 20 bovine *P. multocida* were assigned into four categories of MLST genotypes (ST65, ST79, ST80, ST122). For these genotypes, ST122 (60.00%) was the most dominant. For the 56 porcine *P. multocida*, a total of eight categories of MLST genotypes (ST7, ST9, ST13, ST27, ST50, ST74, ST122, ST287) were present, and ST50 (37.50%), ST13 (23.21%), and ST74 (21.43%) were the common MLST genotypes. The 17 rabbit *P. multocida* were assigned into five categories of MLST genotypes (ST9, ST27, ST50, ST204, and ST298), and ST9 (52.94%) was the most prevalent (see Table 1 and Image 3 in Supplementary Materials).

**Figure 2:**
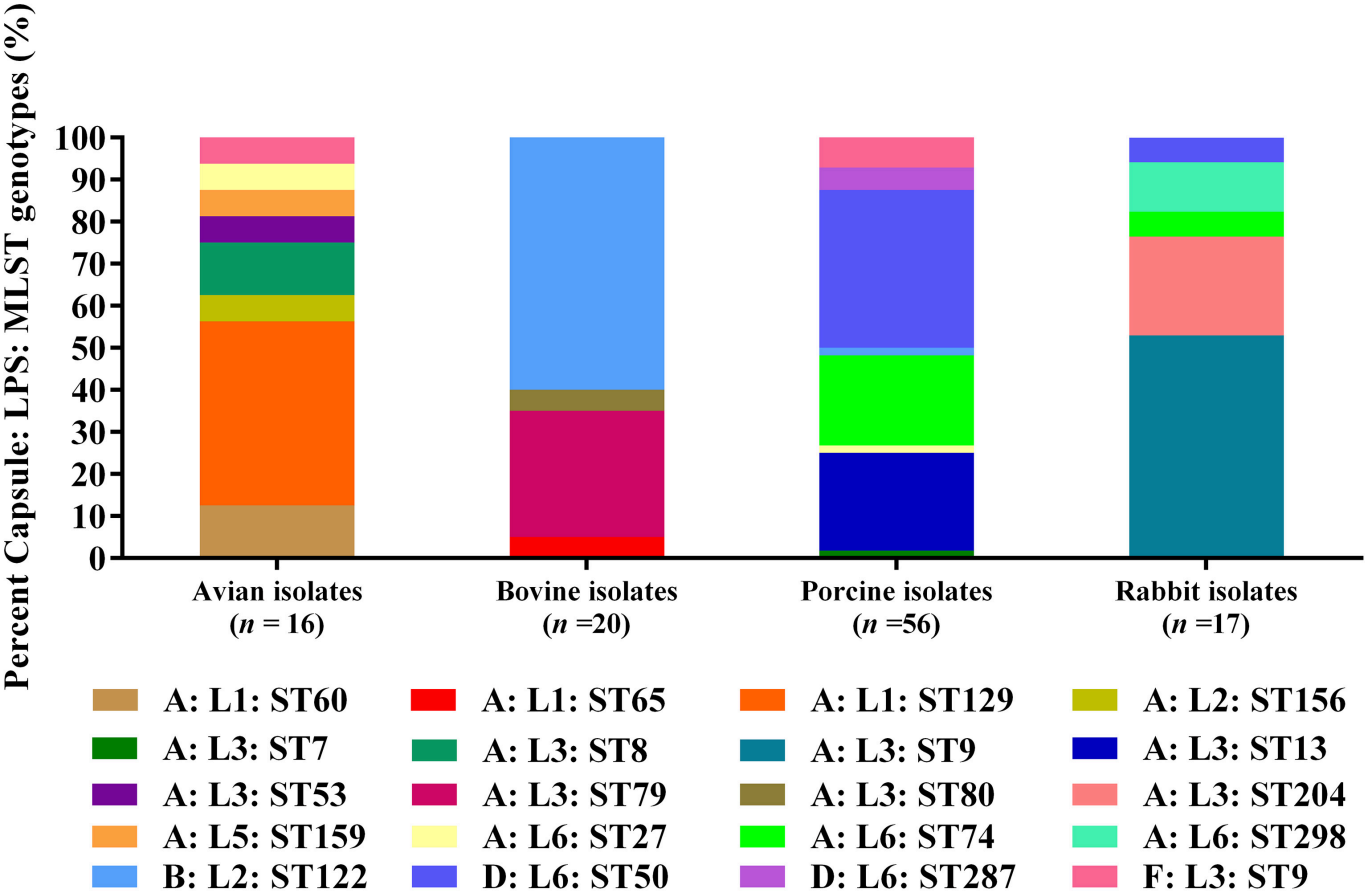
Distribution of different capsule: LPS: MLST genotypes in *P. multocida* isolated from different hosts.

When combining MLST genotypes with capsular types and LPS genotypes, the results showed that a capsular: LPS: MLST genotype A: L1: ST129 (43.75%) was predominant among the 16 avian isolates; and B: L2: ST122 (60.00%) as well as A: L3: ST79 (30.00%) were predominate among the 20 bovine isolates. For the 56 porcine isolates, D: L6: ST50 (37.50%) was the predominate genotype; while for the 17 rabbit isolates, A: L3: ST9 (76.47%) was the predominate (Figure 2).

### Phylogeny

**Figure 3:**
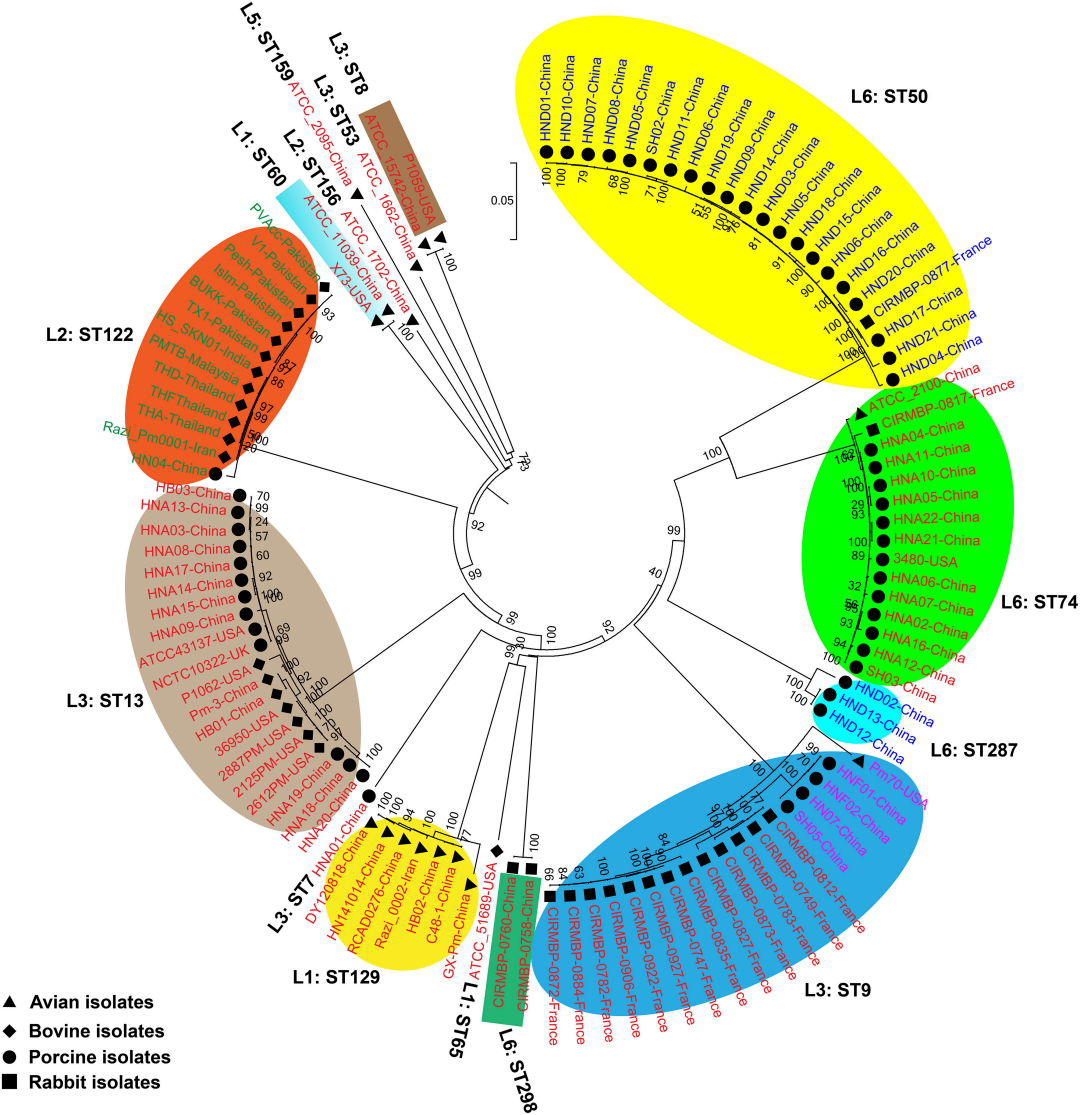
Phylogenetic tree generated based on the SNPs across the whole genome sequences of the *P. multocida* comparing against the reference *P. multocida* ATCC 43137 genome. *P. multocida* capsular type A strains were marked in red, type B strains were marked in green, type D strains were marked in blue, and type F strains were marked in purple. Avian isolates are marked with “
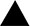
”; Bovine isolates are marked with “
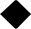
”; Porcine isolates are marked with “
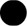
”; Rabbit isolates are marked with “
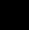
”.

**Figure 4:**
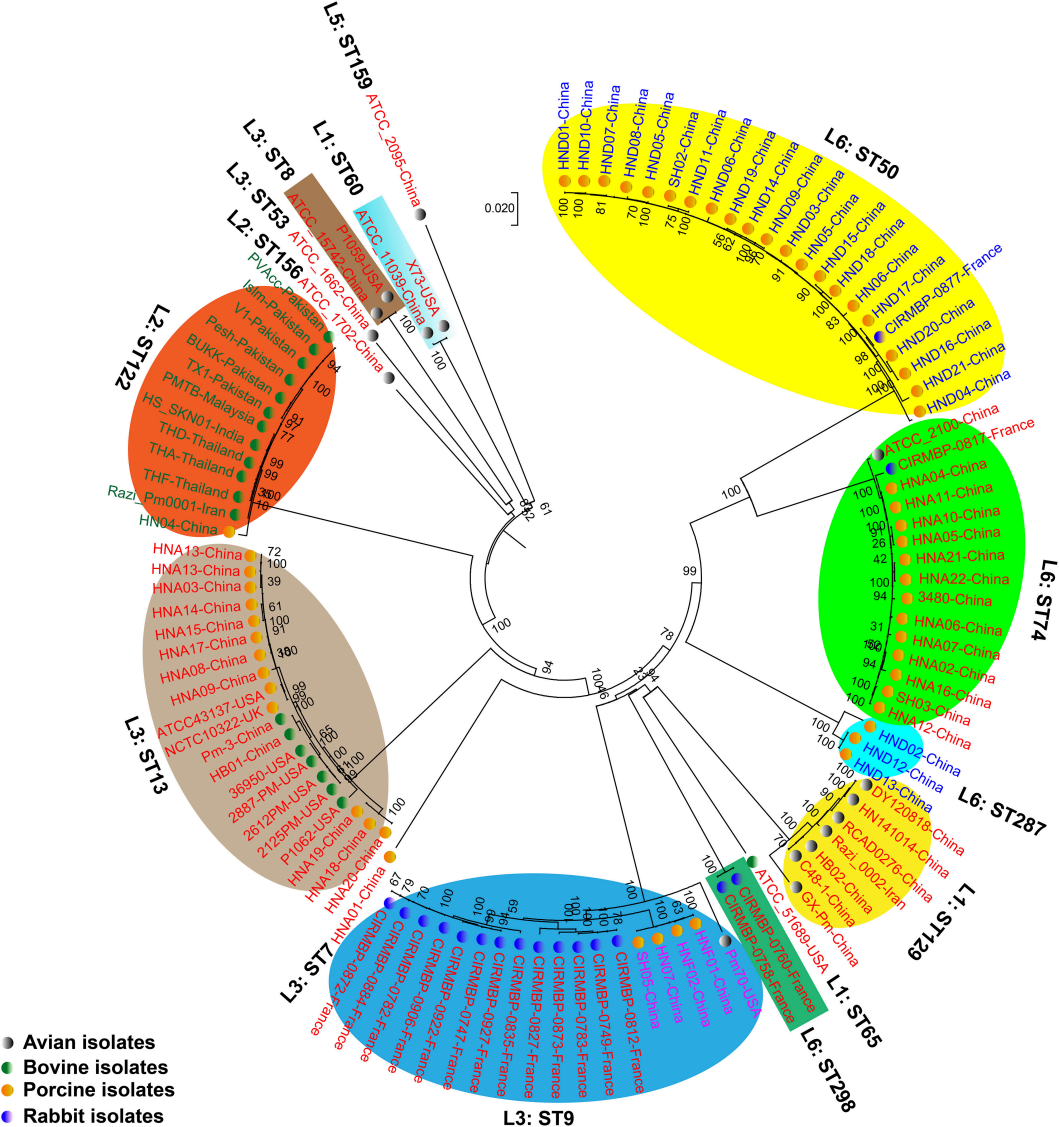
Phylogenetic tree generated based on the SNPs within the single copy core genes among the comparison *P. multocida* genomes. *P. multocida* capsular type A strains were marked in red, type B strains were marked in green, type D strains were marked in blue, and type F strains were marked in purple. Avian isolates are marked with “
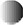
”; Bovine isolates are marked with “
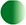
”; Porcine isolates are marked with “
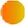
”; Rabbit isolates are marked with “
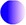
”.

We generated two phylogenetic trees among the *P. multocida* isolates from different isolates collected in this study, and they were based on two criteria, respectively. The first tree was generated using 21,765 SNPs across the WGSs of the *P. multocida* comparing against the reference *P. multocida* ATCC 43137 genome. Using this strategy, it is clearly showed that *P. multocida* strains with the same LPS plus MLST genotypes were phylogenetically clustered together (Figure 3). The second tree was constructed based on the SNPs within all single-copy core genes among the comparison genomes. Based on this tree, *P. multocida* strains with the same LPS plus MLST genotypes were also phylogenetically clustered together (Figure 4).

### Core and pan-genome analysis

**Figure 5:**
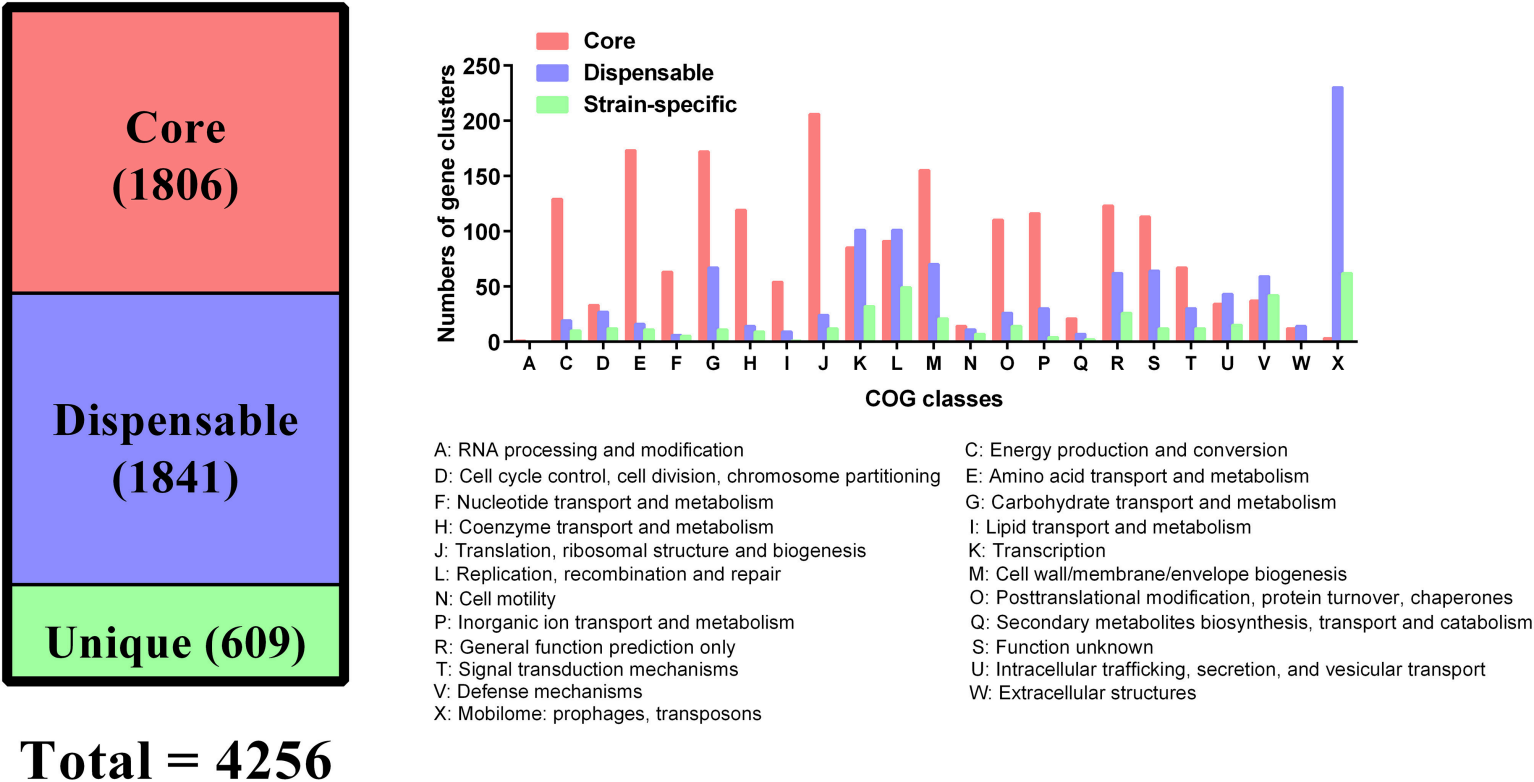
Distribution of core and dispensable genes among the 109 *P. multocida* and their COG functions.

All genes encoded by the 109 *P. multocida* genomes were used for the determination of homologous and unique genes. These analyses identified a shared set of 1,806 genes (core genes), 1,841 dispensable genes, and 609 strain-specific genes (Figure 5). According to COG functional prediction, the core genes mainly participated in translation, ribosomal structure and biogenesis (J), amino acid transport and metabolism (E), carbohydrate transport and metabolism (G), cell wall/membrane/envelope biogenesis (M), energy production and conversion (C), coenzyme transport and metabolism (H), inorganic ion transport and metabolism (P), and posttranslational modification, protein turnover, chaperones (O); while the dispensable genes mainly involved in mobilome (prophages, transposons) (X), replication, recombination and repair (L), and transcription (K); the strain-specific genes mainly participated in mobilome (X), replication, recombination and repair (L), defense mechanisms (V), and transcription (K) (Figure 5).

To figure out genes carried by *P. multocida* genome that are associated with host predilection, we first defined a host predilection related gene as one present among the strains from one host species but absent in strains from all the other host species. This criterion found that there were no genes specific to any hosts. Therefore, we next defined a host predilection related gene was determined as one gene present among 90% of the strains from one host species but absent in 90% of the strains from all the other host species. However, this criterion also found that there were no genes specific to any hosts.

### Distribution of virulence factor-associated genes

A total of 432 putative VFGs were identified by performing BLAST analysis of the 109 *P. multocida* genome sequences against the VFDB database, and these VFGs were assigned into 366 VFG categories (see Table S1 in Supplementary Materials). According to BSR analysis, the distribution of VFGs between different *P. multocida* strains was mainly reflected on the genes responsible for the synthesis of capsule, LPS and fimbrial low-molecular-weight proteins; however, none of these genes were specific to any hosts (see Image 4 in Supplementary Materials). Interestingly, some VFGs displayed a certain level of LPS: MLST genotype-preference. For example, the hyaluronidase encoding gene *pmHAS* was absent in the genotypes L6: ST50, L6: ST287, and L2: ST122 strains; the surface fibrils encoded by the gene *hsf-2* was missing in genotypes L6: ST50 and L3: ST13 strains (see Image 5 in Supplementary Materials).

## Discussion

Birds, pigs, cattle and buffaloes, as well as rabbits are the natural hosts of *P. multocida* (Wilkie et al., 2012; Wilson and Ho, 2013). In this study, we genotyped *P. multocida* strains from these host species using their WGS data. Types A: L1 (56.25%) and A: L3 (18.75%) were the common capsule: LPS genotypes for avian *P. multocida*. It is widely documented that *P. multocida* strains causing avian pasteurellosis (fowl cholera) are most frequently designated Carter: Heddleston serotypes A:1, A:3, or A:4 (Adler et al., 1999; Wilson and Ho, 2013), and *P. multocida* Heddleston serotype 1 and 3/4 strains produced LPS genotypes L1 and L3, respectively (Harper et al., 2011, 2013). Therefore, our results are in agreement with those previously documents (Adler et al., 1999; Wilson and Ho, 2013). It has been noted that *P. multocida* Carter: Heddleston serotype B: 2 strains are commonly responsible for bovine haemorrhagic septicaemia while serovar A: 3 strains are associated with bovine respiratory diseases (Welsh et al., 2004; Dey et al., 2007; Shivachandra et al., 2011; Lainson et al., 2013; Wilson and Ho, 2013). Both haemorrhagic septicaemia and respiratory diseases including pneumonia are the common infections associated with *P. multocida* in bovine species (bovine pasteurellosis) (Wilson and Ho, 2013). The *P. multocida* Heddleston serotype 2 strains produced LPS genotypes L2 (St Michael et al., 2009), and Heddleston serotype 3 strains produced LPS genotype L3 (Harper et al., 2013). This is why genotypes B: L2 (60.00%) and A: L3 (35.00%) possess the first two ranks among the *P. multocida* strains of bovine source. The predominate capsule: LPS genotypes defined among the porcine isolates were types D: L6 (42.86%), A: L3 (25.00%), and A: L6 (23.21%). Those are because *P. multocida* strains with porcine pasteurellosis are frequently designated capsular types A and D (Tang et al., 2009; Liu et al., 2017; Peng et al., 2018), and LPS serovars 3, 4, and 12 (Pijoan et al., 1983; Lainson et al., 2002; Jamaludin et al., 2005). In particular, these results are in agreement with the latest molecular epidemical data in China (Peng et al., 2018). Interestingly, the capsular: LPS genotype of an isolate (strain HN04) from a swine haemorrhagic septicaemia case was B: L2. This finding suggests this type of *P. multocida* might also be able to cause haemorrhagic septicaemia in pigs. The mostly common capsule: LPS genotype identified in rabbit *P. multocida* isolates was A: L3 (76.47%). This result is similar to the previous publication (Townsend et al., 2001), as genotype A: L3 corresponds to *P. multocida* Carter: Heddleston serotype A: 3 or A: 4 (Harper et al., 2013).

The predominate MLST genotype of *P. multocida* from avian species was ST129 (43.75%), and this result is in agreement with Wang et al. (2013). The difference between the two studies is that Wang *et al*. only defined ST129 from the avian *P. multocida* strains but we identified multiple MLST genotypes in addition to ST129; other MLST genotypes such as ST8, ST9, ST27, ST53, ST60 are also common in avian *P. multocida* (Subaaharan et al., 2010). These findings suggest the prevalence of *P. multocida* genotypes from different geographic regions might be different. ST122 was the common MLST genotype in *P. multocida* isolates from bovine species (Table 1). This genotype is widely documented to be associated with bovine haemorrhagic septicaemia, a common pasteurellosis in bovine species (Hotchkiss et al., 2011). Interestingly, the MLST genotype of the porcine isolate HN04 from swine haemorrhagic septicaemia case was also ST122, suggesting that ST122 might be particularly associated with the disease other than the host species. The mostly common MLST genotypes of *P. multocida* from pigs defined in this study were ST50 (37.50%), ST13 (23.21%), and ST74 (19.64%). These results are quite different from the date of a recent molecular epidemiological study (Peng et al., 2018), but this could be explained by the two studies using two different MLST databases to determine the MLST genotypes. When we used the Multiple host MLST database to re-assigned these strains, we found that all ST50, ST13, and ST74 strains were re-assigned as ST11, ST3, and ST10, respectively. These results are in agreement with the latest epidemical data from China (Peng et al., 2018), and also in agreement with the data from Spain (García-Alvarez et al., 2017).

The predominate MLST genotype in *P. multocida* isolates from rabbit species was ST9 (76.47%), which was re-assigned as ST12 using the Multiple host MLST database. Previous studies have documented that ST12 was the common MLST genotype in rabbit *P. multocida* (García-Alvarez et al., 2015; Massacci et al., 2018). Interestingly, a previous study documented that the ST11 (assigned using Multiple host MLST) clone of *P. multocida* is widely spread among farmed rabbits in the Iberian Peninsula and demonstrates respiratory niche association (García-Alvarez et al., 2015). However, the percentage of the ST11 clone (defined as ST50 using RIRDC MLST) defined in this study was very low, only 5.88% (one isolate). According to NCBI genome database (https://www.ncbi.nlm.nih.gov/genome/genomes/912), the WGSs data from the 17 rabbit *P. multocida* using in this study are from France, these findings also suggest that the prevalence of *P. multocida* genotypes in different geographic regions might be different.

When combining the capsular genotypes, LPS genotypes and the MLST genotypes, it is interesting to see that although some *P. multocida* strains from different hosts share the same capsule: LPS genotypes, their MLST genotypes are quite different. The MLST genotypes of *P. multocida* capsule: LPS genotype A: L1 strains from avian species were ST129 and ST69, but the MLST genotype of those type A: L1 strains from bovine species was ST65 (Table 1). However, *P. multocida* strains with the same capsule: LPS: MLST genotype are able to spread in different host species but cause similar disease symptoms. For example, the capsule: LPS: MLST genotype B: L2: ST122 strain is associated with both bovine haemorrhagic septicaemia and swine haemorrhagic septicaemia; the F: L3: ST9 clone can lead to both avian respiratory disease and porcine respiratory disease; the D: L6: ST50 clone is associated with both the porcine respiratory disease and the rabbit respiratory disease (Table 1).

Although many publications have documented that the infection of *P. multocida* isolates displays host predilection (Kubatzky, 2012; Wilkie et al., 2012; Wilson and Ho, 2013), these analyses using comparative genomics found that there were no genes or VFGs specific to any isolates for any specific type of host (see Image 4 in Supplementary Materials). Though the dermonecrotic toxin encoding gene *toxA* was only present in one isolate from pigs (see Image 5 in Supplementary Materials), this gene has been also identified in *P. multocida* isolates from bovine species, avian species, small ruminants, sheep, and goats (Ewers et al., 2006; Sarangi et al., 2016; Shirzad Aski and Tabatabaei, 2016). These findings suggest that *toxA* is not associated with host predilection. Interestingly, it is documented that the presence of *toxA* displays a significant association to the disease status in swine, even though it is detected in *P. multocida* isolates from small ruminants, cattle, and poultry as well (Ewers et al., 2006). This suggests that the disease symptoms caused by *P. multocida* with specific VFGs might be also associated with the host itself and/or the locations that the bacteria are found on the host. However, more studies are necessary for further confirmation. Similar to *toxA*, the transferrin binding protein A encoding gene *tbpA* was also present in isolates from bovine species (see Image 5 in Supplementary Materials). However, this gene has been also reported to be identified in *P. multocida* isolates from ovine species and rabbits (Ewers et al., 2006; Ferreira et al., 2012). These findings suggest that *tbpA* is also not host specific.

It has been proposed that there is little or no correlation between the phylogenetic groups and phenotypic characteristics (Boyce et al., 2012; Moustafa et al., 2015; Cao et al., 2017). However, in this study, using both the SNPs across the WGSs and the SNPs within all single-copy core genes among the comparison genomes, we found that *P. multocida* isolates with the same LPS genotype and MLST genotype could be phylogenetically grouped, though these *P. multocida* strains were isolated from different geographic regions or host species, and/or associated with different disease symptoms (Figures 3, 4). While more data are needed for the further confirmation, these findings suggest the combining both the LPS and MLST typing schemes might better explain the topology seen in the *P. multocida* phylogeny.

In conclusion, we performed whole genome sequencing on *P. multocida* strains isolated from pigs and determined the genetic characteristics of *P. multocida* isolates from avian species, bovine species, pigs and rabbits in this study. Genotyping using WGSs data suggest that *P. multocida* isolates from different host species displayed a certain preference for “capsule/LPS/MLST genotypes.” Although host predilection in *P. multocida* infections are widely documented, our analyses found there were no genes specific to any hosts. In addition, our phylogenetic analyses found that *P. multocida* isolates with the same LPS genotype and MLST genotype could be phylogenetically grouped, which suggests that the combining both the LPS and MLST typing schemes might better explain the topology seen in the *P. multocida* phylogeny.

## Author contributions

ZP, WL, RZ, HC, and BW participated in the conception and design of the work; ZP, WL, ZfX, ZhX, and ZL performed whole genome sequencing and data analysis; FW and LH contributed to bacterial genomic DNA isolation and purification; ZP wrote the manuscript; RZ, HC, and BW helped to revised the manuscript. All authors read and approved the final manuscript.

### Conflict of interest statement

The authors declare that the research was conducted in the absence of any commercial or financial relationships that could be construed as a potential conflict of interest.

## Supplementary material

Table S1 Virulence factor-associated genes of the 109 *P. multocida* isolates predicted by the VFDB database.

Image 1 Distribution of different capsule genotypes in *P. multocida* isolated from different host species.

Image 2 Distribution of different LPS genotypes in *P. multocida* isolated from different species.

Image 3 Distribution of different MLST genotypes in *P. multocida* isolated from different species.

Image 4 Heat-map showing the distribution of virulence factor-associated genes among *P. multocida* isolates from different host species.

Image 5 Heat-map showing the distribution of the 27 main kinds of virulence factor-associated genes among *P. multocida* isolates from different host species.
