# Supplementary figures and images for: Genetic and Phylogenetic Characteristics of Pasteurella multocida Isolates From Different Host Species
Source: Front Microbiol. 2018 Jun 26;9:1408. doi: 10.3389/fmicb.2018.01408 (PMC6029419; doi:10.3389/fmicb.2018.01408)

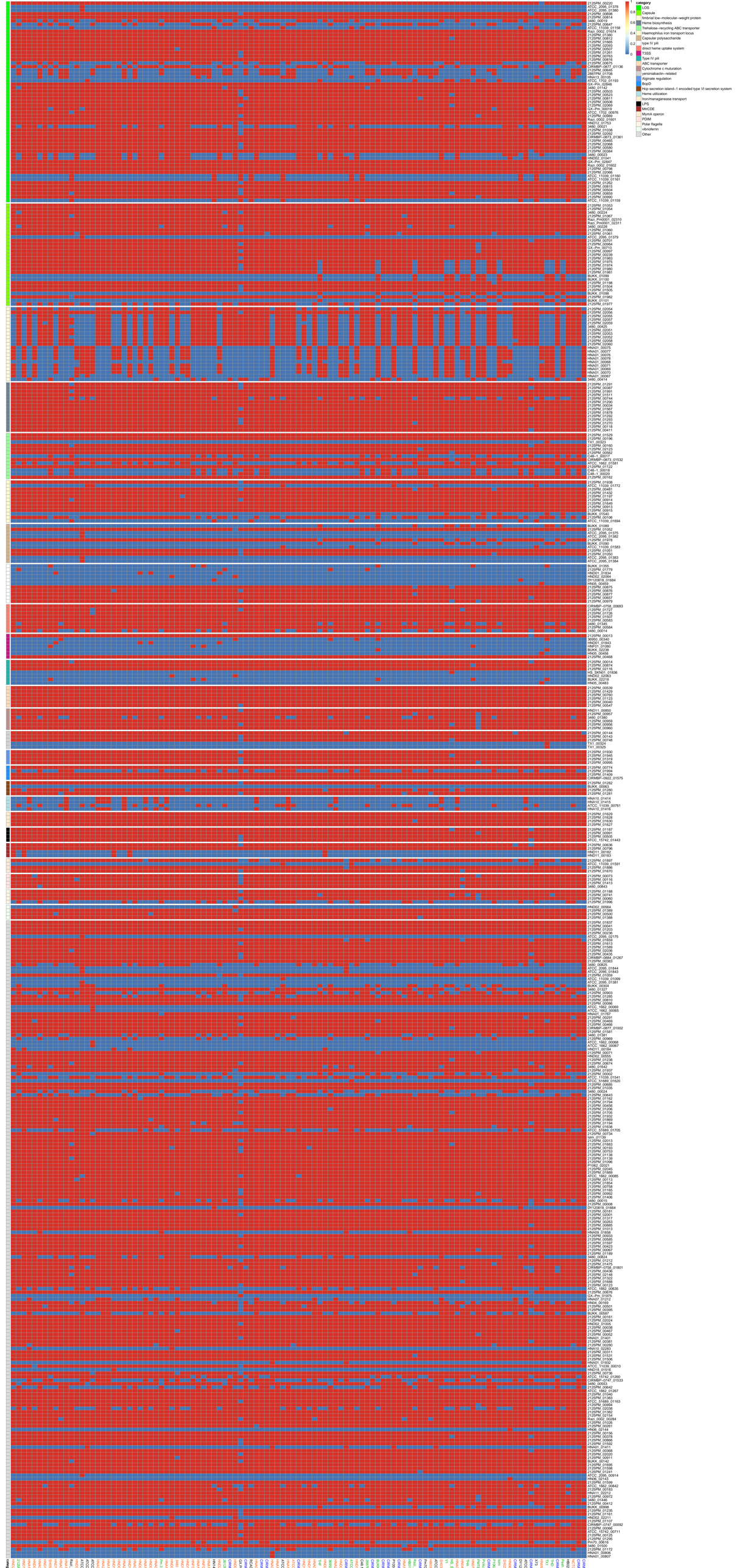

Supplement: Image 4 — Heat-map showing the distribution of virulence factor-associated genes among P. multocida isolates from different host species. [file Image_4.PDF]

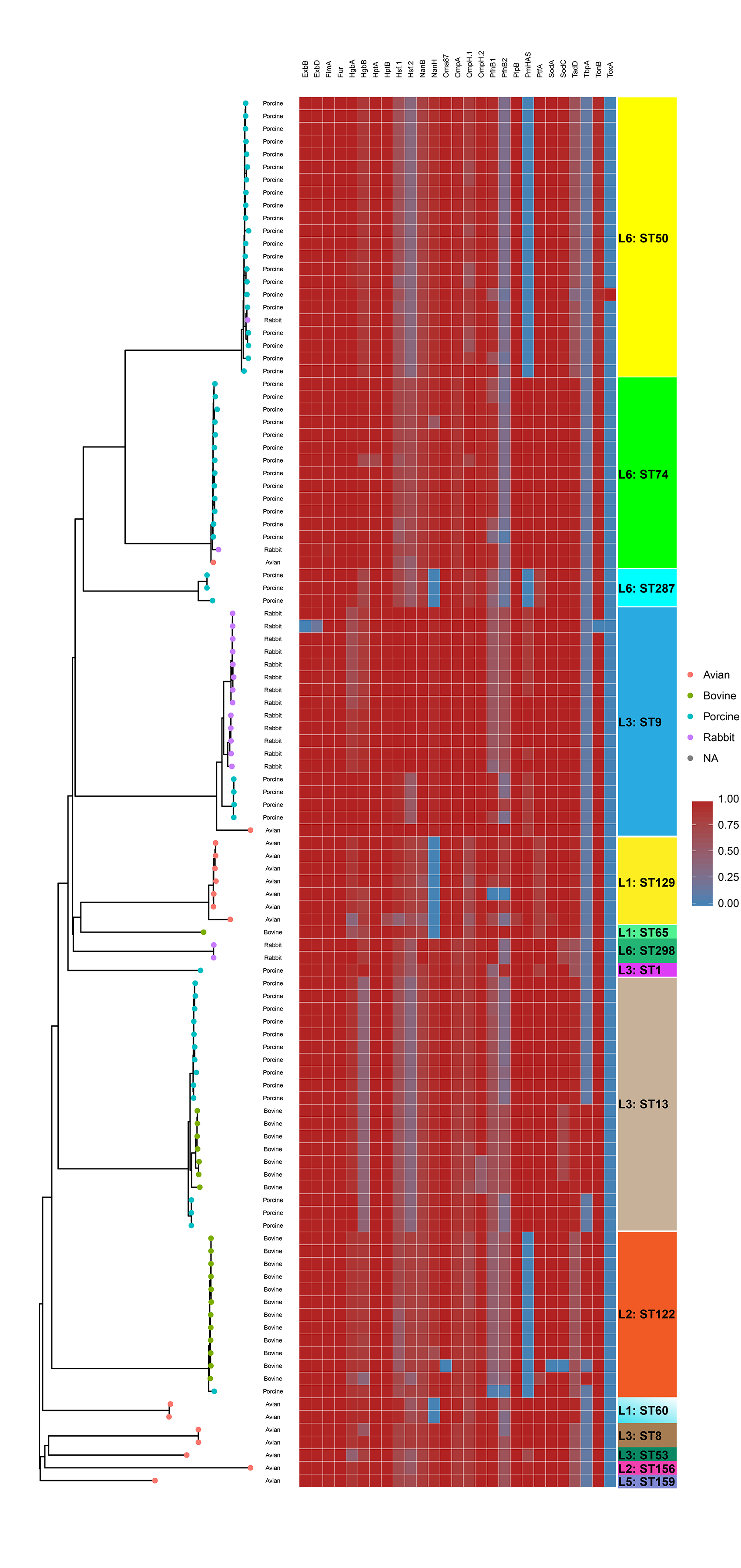

Supplement: Image 5 — Heat-map showing the distribution of the 27 main kinds of virulence factor-associated genes among P. multocida isolates from different host species. [file Image_5.TIF]
